# Mitochondrial Liver Toxicity of Valproic Acid and Its Acid Derivatives Is Related to Inhibition of α-Lipoamide Dehydrogenase

**DOI:** 10.3390/ijms18091912

**Published:** 2017-09-06

**Authors:** Alexei P. Kudin, Hafiz Mawasi, Arik Eisenkraft, Christian E. Elger, Meir Bialer, Wolfram S. Kunz

**Affiliations:** 1 Department of Epileptology and Life & Brain Center, University of Bonn, Sigmund-Freud-Str. 25, D-53105 Bonn, Germany; alexei_kudin@yahoo.com (A.P.K.); christian.elger@ukbonn.de (C.E.E.); 2 Institute for Drug Research, School of Pharmacy, Faculty of Medicine, The Hebrew University of Jerusalem, Jerusalem 91120, Israel; hmawasi@ekmd.huji.ac.il (H.M.); meirb@ekmd.huji.ac.il (M.B.); 3 Institute for Research in Military Medicine, Faculty of Medicine, The Hebrew University of Jerusalem, Jerusalem 91120, Israel; arik@thoraxs.com

**Keywords:** valproic acid, analogues of valproic acid, liver toxicity, metabolic epilepsy, mitochondrial epilepsy

## Abstract

The liver toxicity of valproic acid (VPA) is an established side effect of this widely used antiepileptic drug, which is extremely problematic for patients with metabolic epilepsy and particularly epilepsy due to mitochondrial dysfunction. In the present report, we investigated the reason for liver mitochondrial toxicity of VPA and several acid and amide VPA analogues. While the pyruvate and 2-oxoglutarate oxidation rates of rat brain mitochondria were nearly unaffected by VPA, rat liver mitochondrial pyruvate and 2-oxoglutarate oxidation was severely impaired by VPA concentrations above 100 µM. Among the reactions involved in pyruvate oxidation, pyruvate transport and dehydrogenation steps were not affected by VPA, while α-lipoamide dehydrogenase was strongly inhibited. Strong inhibition of α-lipoamide dehydrogenase was also noted for the VPA one-carbon homolog *sec*-butylpropylacetic acid (SPA) and to a lesser extent for the VPA constitutional isomer valnoctic acid (VCA), while the corresponding amides of the above three acids valpromide (VPD), *sec*-butylpropylacetamide (SPD) and valnoctamide (VCD) showed only small effects. We conclude that the active inhibitors of pyruvate and 2-oxoglutarate oxidation are the CoA conjugates of VPA and its acid analogues affecting selectively α-lipoamide dehydrogenase in liver. Amide analogues of VPA, like VCD, show low inhibitory effects on mitochondrial oxidative phosphorylation in the liver, which might be relevant for treatment of patients with mitochondrial epilepsy.

## 1. Introduction

**Figure 1:**
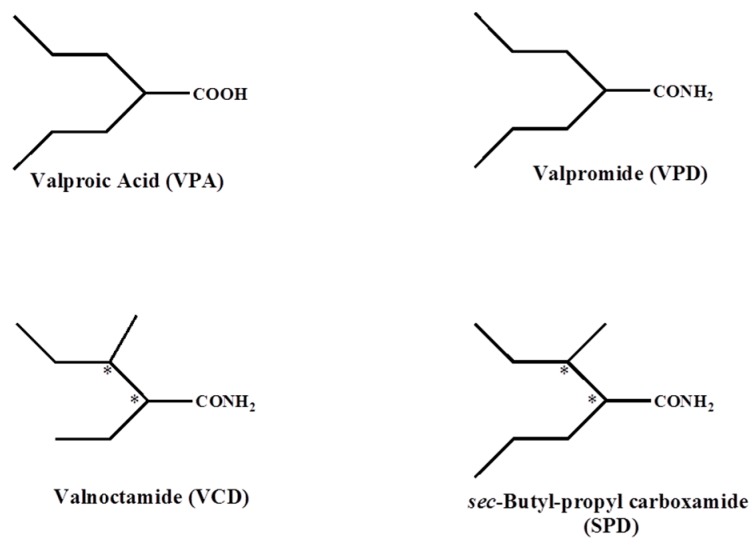
Chemical structures of central nervous system (CNS)-active derivatives of valproic acid. The stars indicate the stereogenic centers of VCD and SPD.

Valproic acid (VPA; Figure 1) is a widely used classical antiepileptic drug with a high responder rate especially in patients with genetic generalized epilepsies (GGE, former nomenclature—idiopathic generalized epilepsies). One well known side effect of VPA is its pronounced liver toxicity, which is relevant in genetic epilepsies affecting the brain and also the liver. One typical example of an epileptic disorder when the liver toxicity of VPA is extremely relevant is Alpers-Hüttenlocher syndrome caused by mutation in mitochondrial DNA-polymerase γ [1,2]. At present, the detailed reason for VPA’s strong liver toxicity is not very well established, but a strong inhibition of pyruvate oxidation of liver mitochondria by VPA has been noted [3,4]. Current concepts propose sequestration of mitochondrial coenzyme A (CoA) [5,6,7], inhibition of pyruvate transport [8], inhibition of ATP- and GTP-dependent succinate:CoA ligases [9], inhibition of hepatic *N*-acetylglutamate synthase [10], and inhibition of α-lipoamide dehydrogenase [11].

To avoid the side effects including liver toxicity and teratogenicity and to retain its wide antiepileptic spectrum of efficacy of the drug, several derivatives of VPA have been developed. The amide of the chiral constitutional isomer of valpromide (VPD; Figure 1; the amide of VPA) valnoctamide (VCD; Figure 1) is a central nervous system (CNS)-active drug [12,13,14] that shows a low biotransformation to the corresponding acid-valnoctic acid (VCA) [12,15,16]. This is different from VPD, which undergoes rapid transformation to VPA in humans. From 1964 until 2005 in several European countries, VCD (racemate) was commercially available as the anxiolytic drug Nirvanil^®^ [13,17]. In its chemical structure, VCD has two stereogenic centers (Figure 1). The mixture of both stereoisomers of VCD (racemate) has been reported to have a wide spectrum of anticonvulsant activity at concentrations that are 2–16 times lower than VPA and are dependent on the epilepsy model [13]. When intraperitoneally administered at seizure onset, 65 mg/kg VCD protected against pilocarpine-induced status epilepticus (SE) in the rat model [18]. However, this protection was lost when administered at 80 mg/kg 30 min after seizure onset [19], but it did block the pilocarpine-induced electrographic SE at the higher dose of 180 mg/kg [20,21]. In contrast to VPA, racemic-VCD, its corresponding acid VCA, and two of its individual stereoisomers, (2*R*,3*S*)-VCD and (2*S*,3*S*)-VCD, did not show any significant teratogenic effects in SWV/Finn mice—an inbred mouse strain that is highly susceptible to VPA-induced teratogenicity [22,23]. *sec*-Butylpropylacetamide (SPD, Figure 1) is a one-carbon homologue of valnoctamide (VCD, Figure 1). SPD has been recently reported by us to show a unique and broad-spectrum antiepileptic profile, which is better (lower ED_50_ values) than VPA and even superior to VCD in some epilepsy models [19,20]. Additionally, SPD blocked the behavioral and electrographic SE induced by the muscarinic agonist pilocarpine, and the organophosphates soman and paraoxon and showed in vivo neuroprotection that was associated with cognitive sparing [19,20,24,25].

In the present work, we investigated in detail the toxic effects of VPA and its corresponding CNS-active analogues on different pathways of oxidative phosphorylation of isolated mitochondria from rat liver and brain. From the obtained IC_50_ values we conclude that the inhibitory effects of CoA conjugates of VPA and its acid analogues are due to inhibition of liver α-lipoamide dehydrogenase.

## 2. Results

### 2.1. The Pyruvate Oxidation of Rat Liver Mitochondria Is Selectively Inhibited by Valproic Acid (VPA) and Its Acid Derivatives

**Figure 2:**
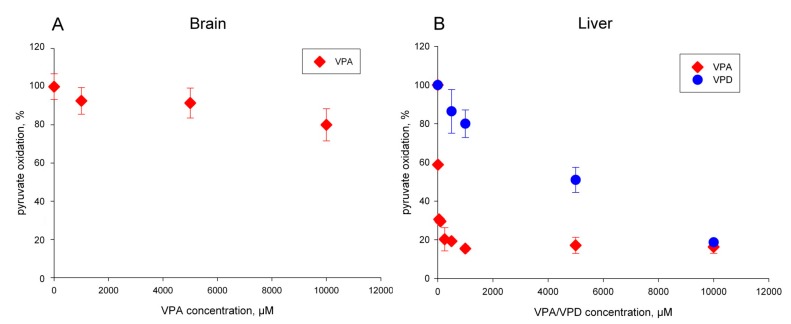
Inhibition of mitochondrial pyruvate oxidation by valproic acid in rat brain mitochondria (**A**) and rat liver mitochondria (**B**). Mitochondria (0.04–0.05 mg protein/mL for brain or 0.2 mg/mL protein/mL for liver) were preincubated for 3 min in presence of 1 mM ATP, 5 mM MgCl_2_ with the indicated amount of VPA. The maximal rate of respiration was determined in presence of 10 mM pyruvate, 5 mM malate, and 1 mM ADP. The plotted rates are averages of three independent experiments. VPD

First, we repeated the observations of previous investigators [3] showing the strong and selective inhibition of ADP-stimulated pyruvate oxidation of isolated rat liver mitochondria by valproic acid with an apparent IC_50_ value of about 50 μM (Figure 2B, red diamonds). In order to achieve stable and reproducible inhibition, we preincubated the mitochondria for 3 min in the presence of the drug and of 1 mM ATP. Interestingly, the same preincubation procedure resulted in almost no inhibition of ADP-stimulated pyruvate oxidation for rat brain mitochondria in a comparable concentration range of VPA (Figure 2A, red diamonds). On the other hand, the amide of valproic acid (VPD) was much less effective in inhibiting the pyruvate oxidation of rat liver mitochondria under similar experimental conditions (Figure 2B, blue circles), having an apparent IC_50_ value of 5 mM. It has been convincingly shown that VPA can be activated by the intramitochondrial medium chain acyl-CoA synthetase to valproyl-CoA, which is then very slowly metabolized by branched chain acyl-CoA dehydrogenase (Acadsb), followed by β-oxidation [26]. Therefore, our experiments support the conclusion that the intramitochondrially formed CoA-conjugate of VPA is essential for toxicity, since amide derivatives of VPA under our experimental conditions cannot form potentially toxic acyl CoA-derivatives. In brain mitochondria, the presence of CoA transferases (which are absent in liver) and the very low expression of intramitochondrial medium chain acyl-CoA synthetase keep the toxic valproyl-CoA concentrations at low levels.

**Figure 3:**
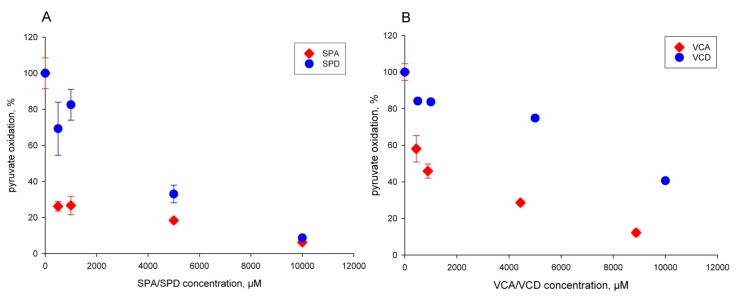
Inhibition of pyruvate oxidation of isolated rat liver mitochondria by derivatives of valproic acid. (**A**) Red diamonds, SPA; blue circles, SPD. (**B**) Red diamonds, VCA; blue circles, VCD. Experimental conditions as indicated in the legend to Figure 2.

In additional experiments, we tested the effects of the other VPA derivatives and their corresponding amides—SPA (*sec*-butylpropylacetic acid) and SPD; VCA and VCD—on their potential to inhibit pyruvate oxidation of rat mitochondria. The acid derivatives were effective, but less potent than VPA—SPA had an IC_50_ of 50 µM, and VCA had an IC_50_ of 900 µM (Figure 3A,B, red diamonds). The corresponding amides (Figure 3A,B, blue circles) were again much less effective in inhibiting pyruvate oxidation.

In further experiments, we tested the potential toxic effects of VPA and its derivatives on ADP-stimulated oxidation rates of octanoyl carnitine, glutamate, and succinate in isolated rat liver mitochondria. As presented in the Figures S1–S3 (Supplementary Materials), we observed only slight inhibitory effects at concentrations of VPA and its derivatives in the range below 1 mM (below 40% inhibition of respiration). That excludes significant toxic effects not directly related to the pyruvate oxidation pathway, like the proposed sequestration of CoA conjugates [5,6,7].

### 2.2. Pyruvate Dehydrogenase and Pyruvate Transport of Rat Liver Mitochondria Are Not Affected by VPA

**Figure 4:**
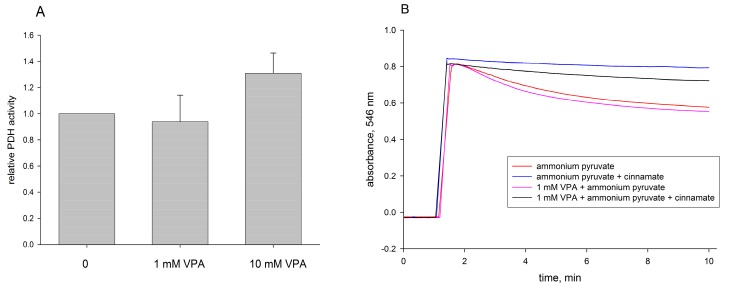
Effect of VPA on pyruvate dehydrogenase activity (**A**) and pyruvate transport activity (**B**) of isolated rat liver mitochondria. Liver mitochondria (2.8–3.6 mg protein/mL) were preincubated for 3 min in presence of 1 mM ATP, 5 mM MgCl_2_ with the indicated amount of VPA. (**A**) The plotted pyruvate dehydrogenase (PDH) activities are averages of three independent experiments; (**B**) Swelling of mitochondria in 0.1 M ammonium pyruvate buffer. One representative experiment (out of 3) is shown. Red trace: control; pink trace: 1 mM VPA; blue trace: 1 mM cinnamate; black trace: 1 mM VPA and 1 mM cinnamate. Experimental conditions as described in Materials and Methods.

In a next series of experiments, we studied which particular reactions involved in pyruvate oxidation might be specifically inhibited by VPA-CoA. The first possibility, already suggested by Turnbull et al. [27], is the dehydrogenation reaction of pyruvate. To evaluate the potential effect of VPA-CoA on this reaction, we preincubated mitochondria with VPA under activating conditions (1 mM ATP present) and then assessed the pyruvate dehydrogenation velocity in the presence of excess α-lipoamide dehydrogenase. As shown in Figure 4A, preincubation of mitochondria with 10 mM VPA and 1 mM ATP resulted even in a slight increase of this particular reaction.

In further experiments, we studied pyruvate transport, which has also been suggested to be responsible for VPA toxicity [8]. To do this, we studied the pyruvate-dependent swelling of mitochondria in ammonium-ion containing medium. This reaction was clearly inhibited by 1 mM cinnamate, a selective inhibitor of pyruvate transport (Figure 4B, blue and black traces), verifying specificity. Pretreatment of mitochondria with 1 mM VPA and 1 mM ATP, however, had no effect on the swelling velocity (Figure 4B, pink trace), indicating the absence of effects on pyruvate transport.

### 2.3. Valproic Acid and Their Acid Derivatives Inhibit α-Lipoamide Dehydrogenase

**Figure 5:**
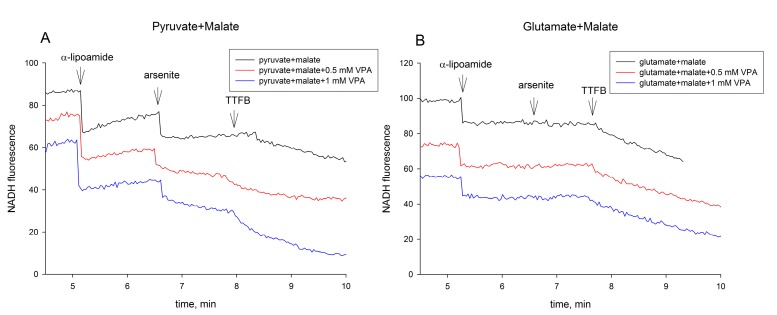
Effect of VPA on pyruvate (**A**) and glutamate (**B**) dependent arsenite-sensitive re-reduction of NAD(P)^+^ by α-lipoamide dehydrogenase. Representative experimental traces of mitochondrial NAD(P)H fluorescence showing re-reduction of NAD(P)^+^ after α-lipoamide (75 µM) additions (first arrow). α-Lipoamide dehydrogenase is blocked by addition of 10 mM arsenite (second arrow). Finally, the uncoupler 4,5,6,7-tetrachloro-2-trifluoromethyl benzimidazole (TTFB, third arrow) was added. The detailed experimental conditions are described in the Materials and Methods section.

In a further set of experiments, we investigated the potential effects of VPA and its derivatives on α-lipoamide dehydrogenase. To do this with isolated mitochondria, we determined the pyruvate-dependent re-reduction of NAD(P)^+^ after the addition of oxidized α-lipoamide (Figure 5A).

**Figure 6:**
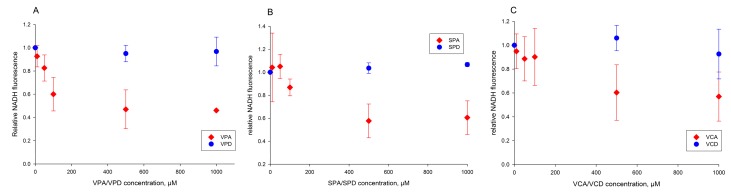
Dose-dependency of effects of VPA and its derivatives on pyruvate-dependent reduction of NAD(P)^+^ by α-lipoamide dehydrogenase. (**A**) Red diamonds, VPA; blue circles, VPD. (**B**) Red diamonds, SPA; blue circles, SPD. (**C**) Red diamonds, VCA; blue circles, VCD. Experimental conditions as indicated in Figure 5.

In the absence of valproic acid, there is a substantial re-reduction of NAD(P)^+^ (black trace), which is strongly inhibited by 0.5 and 1 mM VPA, respectively (red and blue traces). This effect is highly specific for pyruvate (or 2-oxoglutarate) as substrate, since NAD(P)^+^ re-reduction by glutamate after the addition of oxidized α-lipoamide is not observed, and no effects of VPA are detected (Figure 5B). With this assay, we analyzed the inhibitory effects of all VPA derivatives tested in the prior respiration assays with pyruvate as substrate (Figure 6A–C). From the dose dependencies, it can be clearly seen that the sensitivity profile of α-lipoamide dehydrogenase reaction to the VPA derivatives is very similar to the sensitivity profile of pyruvate-dependent oxygen consumption (Table 1, Figure 2B and Figure 3A,B).

Since α-lipoamide dehydrogenase is also involved in the 2-oxoglutate dehydrogenase complex, we tested the sensitivity of ADP-stimulated 2-oxoglutarate oxidation rates of rat liver mitochondria to VPA and its analogues. As shown in the Figure S4A–C (Supplementary Materials), we observed an apparent IC_50_ value of 50 µM under comparable preincubation conditions for VPA and for the acid analogues of VPA—VCA an apparent IC_50_ of 200 µM and for SPA an apparent IC_50_ of 50 µM. These values and the observed lower inhibition effects of the corresponding amides were very similar to the values observed for pyruvate oxidation (Table 1, Figure 2B and Figure 3A,B). Therefore, it can be concluded that the strong inhibitory effects of VPA and its analogues on pyruvate and 2-oxoglutarate oxidation of rat liver mitochondria can be solely explained by inhibition of α-lipoamide dehydrogenase.

## 3. Discussion

The liver toxicity of VPA is one of the most relevant adverse side effects of this broad-spectrum antiepileptic drug. It has been noticed from clinical observations that the liver toxicity of VPA is extremely problematic in Alpers-Hüttenlocher syndrome—a mitochondrial form of epilepsy due to mutations in the mitochondrial DNA polymerase γ affecting brain and liver [1,2,28,29]. However, the detailed reason for the particular exacerbation of this adverse side effect of VPA in certain forms of mitochondrial epilepsy is not clear. To explain this problem, we conducted a detailed study of the in vitro toxicity of VPA and of CNS-active VPA analogues in isolated rat liver mitochondria. Our data clearly show that the pyruvate and 2-oxoglutarate oxidation rates of liver mitochondria are very effectively inhibited by VPA and its acid analogues SPA and VCA (VPA has an IC_50_ values of about 50 µM, SPA of 50 µM, and VCA of 200–900 µM), if the compounds are preincubated in the presence of ATP, allowing the intramitochondrial formation of the corresponding acyl CoA intermediate [4]. Similar to VPA, both SPA and VCA are also very likely activated by intramitochondrial medium chain acyl-CoA synthetase and slowly metabolized by branched chain acyl-CoA dehydrogenase, followed by β-oxidation [26].

Our experimental data are consistent with a selective inhibition of liver α-lipoamide dehydrogenase by valproyl-CoA or the CoA esters of its acid analogues due to the following findings: (i) Pyruvate dehydrogenase and pyruvate transport are almost not affected by preincubation of rat liver mitochondria with VPA in the mM range; (ii) ADP-stimulated octanoylcarnitine and glutamate oxidation of rat liver mitochondria is only slightly affected by preincubation with VPA in the µM range, which excludes considerable CoA sequestration effects; (iii) NAD(P)^+^ re-reduction from α-lipoamide and pyruvate is strongly inhibited by preincubation with VPA and its acid analogues in the µM range; and (iv) both the ADP-stimulated pyruvate and 2-oxoglutarate oxidation rates of rat liver mitochondria are strongly inhibited by preincubation with VPA and its acid analogues in the µM range. The liver specificity of the observed effects—the preincubation of rat brain mitochondria with VPA in the mM range had almost no effect on pyruvate (Figure 2A) and 2-oxoglutarate oxidation rates—appears to be related to the missing activity of CoA transferases in liver mitochondria, which precludes an efficient turnover of slowly metabolizing acyl CoAs. Moreover, intramitochondrial medium chain acyl-CoA synthetases allowing the ATP-dependent formation of CoA conjugates of VPA and its acid analogues have in brain very low expression levels (cf.: http://www.gtexportal.org/home/gene). The presented experimental data cannot exclude potential inhibitory effects of β-oxidation intermediates of VPA and its acid analogues on liver α-lipoamide dehydrogenase. Their concentrations reach only very low levels in comparison to valproyl-CoA [4], which makes this alternative explanation less likely.

The therapeutic blood levels of VPA in patients with epilepsy are in the range of 40–100 mg/L, which corresponds to a concentration range of 277–693 µM [30]. Therefore, the very high sensitivity of liver α-lipoamide dehydrogenase with an apparent IC_50_ of 80µM to VPA could convincingly explain why, under the condition of mitochondrial DNA depletion in the liver of Alpers-Hüttenlocher patients, the already limited capacity of mitochondrial oxidative phosphorylation would be further impaired by strong inhibition of this enzyme affecting both pyruvate dehydrogenase and 2-oxoglutarate dehydrogenase complexes. Other potential reasons for mitochondrial toxicity of VPA, like CoA sequestration CoA [5,6,7], the inhibition of pyruvate transport [8], the inhibition of ATP- and GTP-dependent succinate:CoA ligases [9], and the inhibition of hepatic *N*-acetylglutamate synthase [10], seem to not be relevant, since their apparent IC_50_ values are all the mM range.

It has to be mentioned that our study is mostly relevant for valproate-associated adverse effects in liver reported for mitochondrial forms of epilepsy. Under these circumstances, liver mitochondria with genetically impaired oxidative phosphorylation [28] are additionally challenged by inhibition of α-lipoamide dehydrogenase, which obviously leads to an amplification of toxic effects. It cannot be excluded that, in other forms of epilepsy, there are still additional relevant targets of VPA-associated adverse effects.

Among the tested acid analogues of VPA, VCA showed the lowest mitochondrial toxicity in liver. The corresponding amides—VPD, SPD, and VCD—that are not suitable for a fast, direct conversion to the corresponding CoA intermediate under our in vitro conditions, are much less potent mitochondrial toxins. In this context, it has to be noted that VPD undergoes a rapid biotransformation to VPA under in vivo conditions in humans, while VCD appears to be much more resistant to amidases [16]. Unlike SPD, which is currently in a preclinical stage of development, VCD recently underwent a phase IIb clinical trial in bipolar disorder, but the interim analysis was not successful [24,31]. Nevertheless, due to its metabolic stability (i.e., minimal conversion to its corresponding acid) [16], safety in humans, low liver mitochondrial toxicity (this report), and lack of teratogenicity [24,32], VCD might be a good alternative for VPA in patients with epilepsy, particularly for women of child bearing age and in patients under the risk of severe liver involvement. However, this still remains to be shown in further in vivo experimental studies in animals and in a successful clinical trial in patients.

## 4. Materials and Methods

### 4.1. Materials

VPA and all major chemicals were commercially obtained from Sigma-Aldrich (St. Louis, MO, USA). VCD, VCA, SPD and SPA were synthesized using previously described methods [19,21].

### 4.2. Isolation of Rat Brain and Rat Liver Mitochondria

We isolated mitochondria from a single rat brain using the protocol of Rosenthal et al. [33], which was slightly modified to obtain better characteristics [34]. Rat liver mitochondria were prepared according to Steinbrecht and Kunz [35] with the following modifications. The liver of one 50–60 days old Wistar rat (approx. 5 g) was immediately transferred into ice-cold solution A (0.3 M sucrose, 3 mM EGTA, pH 7.4) and shaken to wash out blood. Then we minced the liver, added around 30 mL of solution A, and homogenized it at 600 units/s using a potter homogenizer (Potter S, B. Braun Melsungen, Melsungen, Germany). Thereafter, the homogenate was centrifuged at 900× *g* for 5 min. The supernatant was passed through a cheesecloth and centrifuged at 12,000× *g* for 10 min. The resulting pellet was dissolved with approx. 20 mL of ice-cold solution B (0.3 sucrose, pH 7.4 adjusted with small amounts of Tris-base). The solution was transferred to a small glass homogenizer and homogenized 10–12 times manually. Finally, the suspension was centrifuged at 12,000× *g* for 10 min, and the resulting pellet was dissolved in the proportion 160 µL solution B per 1 g liver wet weight.

### 4.3. Determination of Substrate Oxidation Rates

Substrate oxidation rates were determined using high resolution respirometry. Rat brain and liver mitochondria (0.04–0.05 mg protein/mL for brain or 0.2 mg/mL protein/mL for liver) were incubated for 3 min in air gassed medium (10 mM KH_2_PO_4_, 60 mM KCl, 60 mM Tris-HCl, 110 mM mannitol, and 0.5 mM EDTA (pH 7.4)) in the presence of 5 mM MgCl_2_, 1 mM ATP and the respective amount of VPA or its analogues at 30 °C. Than the maximal substrate-supported oxygen uptake was measured by high-resolution respirometry using an Oroboros oxygraph [36] in the presence of different substrates and 2 mM ADP.

### 4.4. Determination of Pyruvate Dehydrogenase (PDH) Activity and Measurement of Mitochondrial Pyruvate Uptake

To determine the pyruvate dehydrogenase (PDH) activity and pyruvate transport, rat liver mitochondria (2.8–3.6 mg protein/mL) were incubated in the Oxygraph chamber in MTP medium in the presence of 5 mM MgCl_2_, 1 mM ATP, and the respective amount of VPA or its analogues for 3 min. PDH activity in this suspension was determined according to Scislowski and Davis [37] with the following modifications: the mitochondrial suspension (0.3 mg protein) was placed into a 0.5 mL spectrophotometric cuvette, containing 0.1 M potassium phosphate buffer (pH 7.4), 5 mM MgCl_2_, 3 mM NAD^+^, 0.1 mM CoASH, 2 mM dithiothreitol, 0.1% *N*-dodecyl-β-d-maltoside, 0.4 mM thiamine pyrophosphate, 20 µg/mL diaphorase (Boehringer Mannheim, Mannheim, Germany), and 10 mM pyruvate. PDH activity was measured at 30 °C using a spectrophotometer (Aminco DW 2000, SLM Instruments, Urbana, IL, USA) with 340–380 nm dual wavelength photometry.

For pyruvate transport determinations, the mitochondrial suspension (0.6–0.7 mg protein) was placed into a 3 mL spectrophotometric cuvette, containing 5 mM MgCl_2_, 2 µM rotenone, 1.5 µg/mL antimycin, and 0.1 M ammonium pyruvate (obtained from ammonium hydroxide and pyruvic acid). When indicated, 1 mM cinnamate was added [38]. Mitochondrial swelling was estimated at 30 °C using a spectrophotometer (Aminco DW 2000, SLM Instruments) at 546 nm.

### 4.5. Determination of α-Lipoamide Dehydrogenase Activity in Intact Mitochondria

α-Lipoamide dehydrogenase activity of rat liver mitochondria was analyzed, probing the reversed reaction of the enzyme [39]. After 3 min incubation of rat liver mitochondria (0.3 mg protein/mL) in MTP medium in the presence of 5 mM MgCl_2_, 1 mM ATP, and the respective amounts of VPA (or its derivatives) in 1 mL fluorimetric cuvette, the following additions were subsequently made: 5 mM malate and 10 mM pyruvate, 6.7 µM rotenone, 4 µM KCN, 75 µM α-lipoamide (30 mM stock solution was dissolved in 50% alcohol), and 10 mM arsenite, 0.4 µM 4,5,6,7-tetrachloro-2-trifluoromethyl benzimidazole (TTFB). The NAD(P)H reduction was monitored at λ_ex_ = 340 nm, λ_em_ = 450 nm in a Shimadzu RF-5001PC (Shimadzu Scientific, Columbia, MD, USA) spectrofluorophotometer. DLDH activity was estimated from the NAD(P)H fluorescence increase after the addition of α-lipoamide. In agreement with Luis et al. [11], complete inhibition of DLDH activity was observed after the application of 10 mM arsenite.

## Figures and Tables

**Table 1:** Apparent IC_50_ values of inhibition of different oxidation reactions of isolated rat liver mitochondria by VPA and its analogues.

	VPA	VPD	VCA	VCD	SPA	SPD
pyruvate oxidation	50 µM	5 mM	900 µM	8 mM	50 µM	3 mM
2-oxoglutarate oxidation	50 µM	8 mM	200 µM	5 mM	50 µM	1 mM
glutamate oxidation	10 mM	3 mM	2 mM	4.5 mM	2 mM	2 mM
α-lipoamide dehydrogenase	80 µM	>1 mM ^1^	300 µM	>1 mM ^1^	100 µM	>1 mM ^1^

^1^ Estimated value. VPA: valproic acid; VPD: valpromide; VCA: valnoctic acid; VCD: valnoctamide; SPA: *sec*-butylpropylacetic acid; SPD: *sec*-butylpropylacetamide.

## Supplementary Materials

Supplementary materials can be found at www.mdpi.com/1422-0067/18/9/1912/s1.

## Abbreviations

VPA	Valproic acid
VPD	Valpromide
VCA	Valnoctic acid
VCD	Valnoctamide
SPA	*sec*-Butylpropylacetic acid
SPD	*sec*-Butylpropylacetamide
CNS	Central nervous system
PDH	Pyruvate dehydrogenase
SE	Status epilepticus
