# Supplementary material for: Mitochondrial Liver Toxicity of Valproic Acid and Its Acid Derivatives Is Related to Inhibition of α-Lipoamide Dehydrogenase
Source: Int J Mol Sci. 2017 Sep 6;18(9):1912. doi: 10.3390/ijms18091912 (PMC5618561; doi:10.3390/ijms18091912)
Supplement: Supplementary file 1 [file ijms-18-01912-s001.pdf]

Supplementary Materials: Mitochondrial Liver Toxicity of Valproic Acid and Its Acid Derivatives is Related to Inhibition of α-Lipoamide Dehydrogenase

Alexei P. Kudin, Hafiz Mawasi, Arik Eisenkraft, Christian E. Elger, Meir Bialer and Wolfram S. Kunz

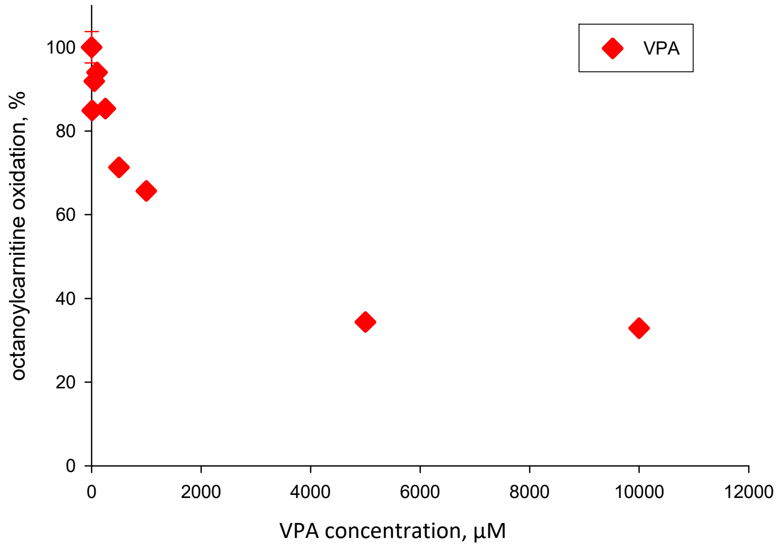

Figure S1. Inhibition of ADP-stimulated octanoylcarnitine (+malate) oxidation rate of rat liver mitochondria by valproic acid (VPA). Mitochondria (0.2 mg/mL protein/mL) were preincubated for 3 min in presence of 1 mM ATP, 5 mM $MgCl_2$ with the indicated amount of VPA. The maximal rate of respiration was determined in presence of 1 mM octanoylcarnitine, 5 mM malate, and 1 mM ADP.

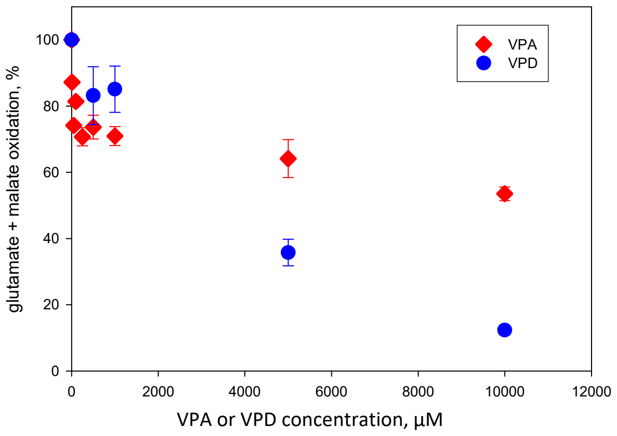

(**a**)

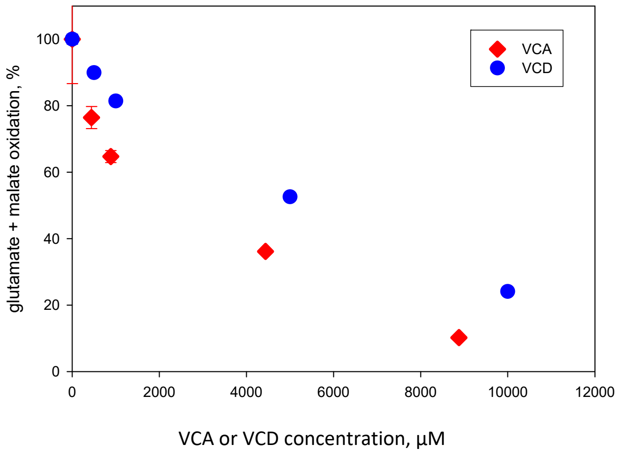

(**b**)

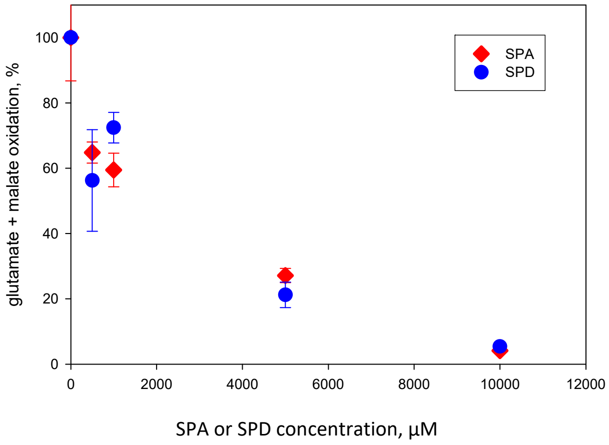

(**c**)

Figure S2. Inhibition of ADP-stimulated glutamate + malate oxidation rate of rat liver mitochondria by VPA, valpromide (VPD) (**A**), valnoctic acid (VCA), valnoctamide (VCD) (**B**) and *sec*-butylpropylacetic acid (SPA), *sec*-butylpropylacetamide (SPD) (**C**). Mitochondria (0.2 mg/mL protein/mL) were preincubated for 3 min in presence of 1 mM ATP, 5 mM $MgCl_2$ with the indicated amount of the drugs. The maximal rate of respiration was determined in presence of 10 mM glutamate, 5 mM malate, and 1 mM ADP. The plotted rates are averages of three independent experiments.

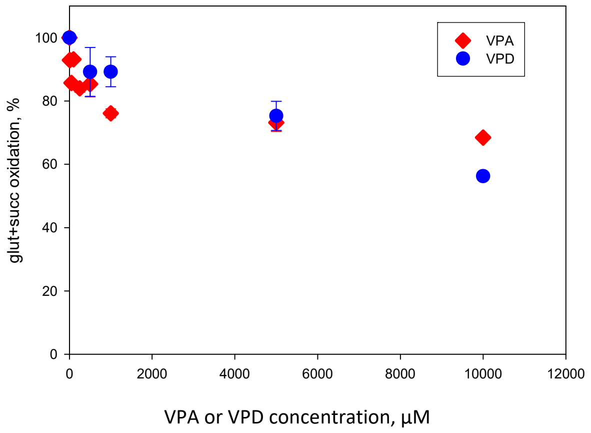

(**a**)

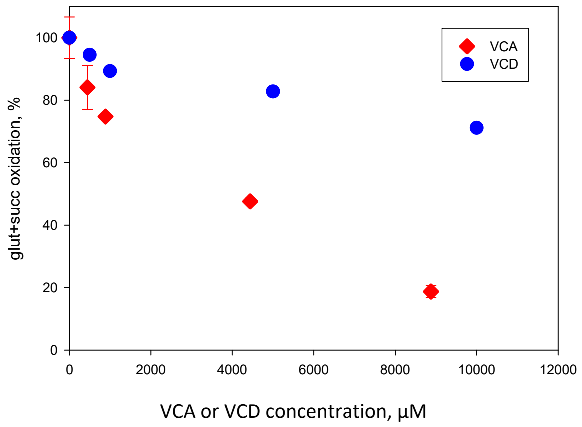

(**b**)

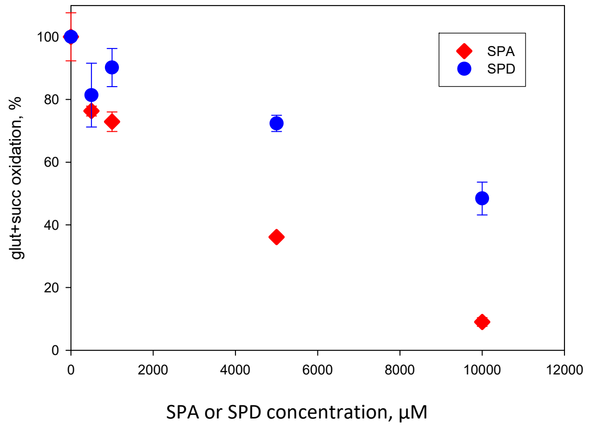

(**c**)

Figure S3: Inhibition of ADP-stimulated glutamate + succinate oxidation rate of rat liver mitochondria by VPA, VPD (**A**); VCA, VCD (**B**) and SPA, SPD (**C**). Mitochondria (0.2 mg/mL protein/mL) were preincubated for 3 min in presence of 1 mM ATP, 5 mM $MgCl_2$ with the indicated amount of the drugs. The maximal rate of respiration was determined in presence of 10 mM succinate, 10 mM glutamate, 5 mM malate, and 1 mM ADP. The plotted rates are averages of three independent experiments.

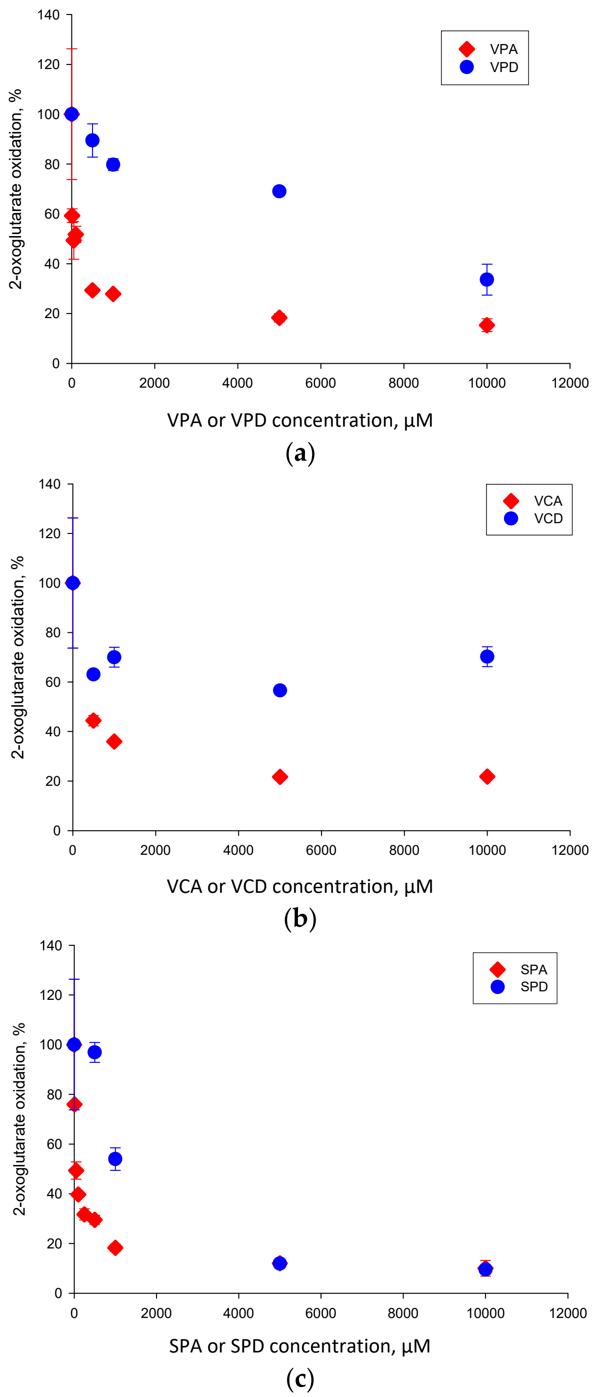

Figure S4. Inhibition of ADP-stimulated 2-oxoglutarate oxidation rate of rat liver mitochondria by VPA, VPD (**A**), VCA, VCD (**B**) and and SPA, SPD (**C**). Mitochondria (0.2 mg/mL protein/mL) were preincubated for 3 min in presence of 1 mM ATP, 5 mM $MgCl_2$ with the indicated amount of the drugs. The maximal rate of respiration was determined in presence of 10 mM 2-oxoglutarate and 1 mM ADP. The plotted rates are averages of 3 independent experiments.
